# Gently does it for submicron crystals

**DOI:** 10.7554/eLife.01662

**Published:** 2013-11-19

**Authors:** Oliver B Zeldin, Axel T Brunger

**Affiliations:** 1 **Oliver B Zeldin** is in the Department of Molecular and Cellular Physiology and the Howard Hughes Medical Institute, Stanford University, Stanford, United Stateszeldin@stanford.edu; 2 **Axel T Brunger** is in the Departments of Molecular and Cellular Physiology, Neurology and Neurological Sciences, Structural Biology, Photon Science and the Howard Hughes Medical Institute, Stanford University, Stanford, United Statesbrunger@stanford.edu

**Keywords:** electron crystallography, electron diffraction, electron cryomicroscopy (cryo EM), method development, protein structure, None

## Abstract

A protein structure has been refined with electron diffraction data obtained by using a very weak electron beam to collect large numbers of diffraction patterns from a few sub-micron-sized three-dimensional crystals.

**Related research article** Shi D, Nannenga BL, Iadanza MG, Gonen T. 2013. Three-dimensional electron crystallography of protein microcrystals. *eLife* **2**:e01345. doi: 10.7554/eLife.01345

**Image** Lysozyme crystals (arrows) that are too small for X-ray crystallography can be studied with electron crystallography.

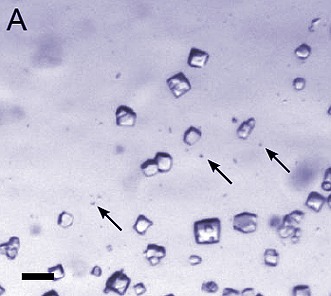

X-ray diffraction from crystals containing billions of identical protein molecules is by far the dominant source of structural information for biological scientists. However, since the X-ray beam damages the crystal, there is a lower limit on how small a single crystal can be and still allow enough data to be collected under cryogenic conditions (Garman, 2010; Holton and Frankel, 2010). Moreover, although about a third of all macromolecules form some kind of crystal (Rupp, 2007), only a small fraction of these crystals are large enough and sufficiently well ordered to allow the macromolecular structure to be determined from a single crystal. New techniques are therefore needed to study the many macromolecules and macromolecular complexes that do not form crystals that are suitable for traditional crystallographic analysis.

Now, in *eLife*, Tamir Gonen and co-workers at the Janelia Farm Research Campus—including Dan Shi, Brent Nannenga and Matthew Iadanza as joint first authors—show that electrons can be used to determine protein structures from sub-micron crystals that are too small for X-ray crystallography at current third-generation synchrotron radiation sources (Shi et al., 2013). As a proof of concept, the Janelia Farm team has used electron diffraction data from sub-micron crystals to refine the structure of lysozyme, a widely used test protein, at a resolution of 2.9 Å. In crystallography, the resolution of the refined structure refers to the minimum spacing (between crystal lattice planes) that was used in the refinement.

From the point of view of crystallography, electrons have various advantages and disadvantages compared with X-rays. Electrons interact with matter much more strongly than X-rays do, so they are diffracted much more than X-rays (typically by several orders of magnitude). This is the reason why electron crystallography can, in theory, be used to determine protein structures from crystals that are too small for X-ray crystallography (Henderson, 1995). However, the strong interactions between electrons and matter, combined with the fact that electrons can sometimes scatter multiple times as they pass through the crystal, makes the analysis of electron diffraction patterns more challenging than the analysis of X-ray diffraction patterns. Moreover, if the crystal is thick, the beam is likely to be absorbed before it penetrates the whole crystal. This means that large three-dimensional (3D) crystals are unsuitable for electron diffraction, and thin samples must be used.

To date, these problems have limited electron crystallography to the small number of systems that can form two-dimensional (2D) crystals, which have fewer unit cells interacting with the beam, and these investigations have yielded several high resolution structures (Wisedchaisri et al., 2011). However, it is difficult and time-consuming to determine protein structures from 2D crystals because electrons are only weakly diffracted by a single layer of unit cells: this means that the electron dose must be high and that the diffraction data must be collected from many regions of the crystal in order to avoid excess damage. Moreover, the resulting data and models have much lower resolution in the direction perpendicular to the unit cell. It is possible to overcome some of these problems by using extremely thin three-dimensional crystals, and diffraction patterns with a resolution of 1.8 Å have been recorded for lysozyme crystals with a thickness of just ∼100 nm (Nederlof et al., 2013). However, these data did not yield an atomic model of the structure.

Gonen and co-workers now show that by using somewhat thicker (∼500 nm) crystals and a very low dose of electrons, it is possible to combine the high signal-to-damage ratio of electron diffraction and the improved diffracting power of 3D crystals (compared to ultrathin 3D crystals) to obtain a reasonably complete diffraction data set. The experiments were performed in a transmission electron cryo-microscope, an instrument that is often used for imaging rather than diffraction, and the electron dose (less than nine electrons per Å^2^ spread over about 90 images) was a factor of 200 below what is considered to be a standard electron dose for imaging applications.

Getting structural information from smaller and smaller crystals is a topic of great interest in structural biology. The development of micro-focus beamlines at third-generation synchrotrons has enabled the collection of entire diffraction datasets from single crystals as small as 15×15×15 μm^3^ (Watson et al., 2012), or from multiple crystals with edges shorter than 5 μm (Ji et al., 2010). An alternative approach is to use an X-ray free electron laser (XFEL) to generate ultrashort (∼50 femtoseconds) pulses of X-rays that contain sufficient photons to generate a diffraction image before any damage can take place (Neutze et al., 2000; Chapman et al., 2011). Using this method, and over a million radiation-damage-free diffraction images from micron-sized lysozyme crystals recorded at the LCLS facility (an XFEL at Stanford), the structure of lysozyme was refined to a resolution of 1.9 Å (Boutet et al., 2012).

The damage-free nature of the XFEL data is extremely attractive and it should be possible, in principle, to collect data from crystals with dimensions as small as about 100 nanometers (Bogan, 2013). However, millions of crystals were needed for these experiments, and reducing this number will be a significant challenge. Moreover, access to XFEL facilities is limited. Since transmission electron cryomicroscopes are available in many laboratories, whereas there are only a handful of XFELs in the world, one possible application of the electron diffraction approach pioneered by the Janelia Farm team would be to pre-screen samples so that only the most promising are investigated further at XFEL facilities.

The proof-of-concept structure by Gonen and co-workers was achieved with comparatively unsophisticated data processing, and this resulted in many diffraction spots at high resolution being discarded: moreover, dynamic scattering effects were not taken into account. As a consequence, the resolution they obtained (2.9 Å) is not as good as the diffraction limit for lysozyme crystals (1.7 Å). New computational methods are needed to address these issues.

It is very encouraging that, despite these caveats, the lysozyme model could be refined to 2.9 Å. Moreover, it is gratifying that the data passed rigorous validation tests, showing that they were not over-fitted during the later stages of data processing. In conclusion, the micro-electron diffraction method has the potential for higher resolution, and the applicability of this method to difficult targets shows great promise.
