# Correction: Gently does it for submicron crystals

**DOI:** 10.7554/eLife.01979

**Published:** 2013-12-03

**Authors:** Oliver B Zeldin, Axel T Brunger

Zeldin OB, Brunger AT. 2013. Gently does it for submicron crystals. *eLife* **2**:e01662. doi: http://dx.doi.org/10.7554/eLife.01662. Published 19 November 2013

The original version of this article contained three errors: the last sentence of the fourth paragraph referred to 100 Å where it should have referred to 100 nm; the first sentence of the fifth paragraph referred to 500 Å where it should have referred to 500 nm; and the last sentence of the fifth paragraph referred to a dose of ‘less than nine electrons per Å^2^’ where it should have referred to a dose of ‘less than nine electrons per Å^2^ spread over about 90 images’.

The article has been corrected accordingly.

